# Influence of the Type of Diet on the Incidence of Pathogenic Factors and Antibiotic Resistance in Enterococci Isolated from Faeces in Mice

**DOI:** 10.3390/ijms20174290

**Published:** 2019-09-02

**Authors:** Beatriz Sánchez, Antonio Cobo, Marina Hidalgo, Ana M. Martínez-Rodríguez, Isabel Prieto, Antonio Gálvez, Magdalena Martínez-Cañamero

**Affiliations:** 1 Área de Microbiología, Departamento de Ciencias de la Salud, Universidad de Jaén, Paraje de Las Lagunillas s/n, 23071 Jaen, Spain; 2 Departamento de Estadística e Investigación Operativa, Universidad de Jaén, Paraje de Las Lagunillas s/n, 23071 Jaen, Spain; 3 Área de Fisiología, Departamento de Ciencias de la Salud, Universidad de Jaén, Paraje de Las Lagunillas s/n, 23071 Jaen, Spain

**Keywords:** enterococci, virulence, antibiotic resistance, olive oil, high fat diets

## Abstract

A comparative study on potential risks was carried out in a collection of 50 enterococci isolated from faeces of mice fed a standard or a high-fat diet enriched with extra virgin olive oil, refined olive oil or butter, at the beginning, after six weeks and after twelve weeks of experiments. Strains were biochemically assessed and genetically characterized. *E. faecalis* and *E. casseliflavus* were the most frequently isolated species in any diet and time points. Apart from the fact of not having isolated any strain from the virgin olive oil group during the last balance, we found statistically significant differences $\left(p<0.05\right)$ among the diets in the percentage of antibiotic resistance and in the presence of the enterococcal surface protein gene (*esp*), as well as a tendency $\left(p<0.1\right)$ for the presence of the tyrosine decarboxylase gene (*tdc*) to increase over time in the group of isolates from the standard diet. When the resistance of the strains to virgin or refined olive oil was studied, only the group of enterococci from high fat diets showed a significantly higher percentage of resistance to refined olive oil $\left(p<0.05\right)$, while both types of oil equally inhibited those isolated from the standard diet $\left(p>0.05\right)$.

## 1. Introduction

The intestinal microbial diversity will be determined by the interactions among the organisms that compose it, the genetics of the host and the diets applied [1]. Diet, in fact, has a marked influence on the intestinal microbiota of the host [2] and much attention has been drawn specifically on high fat diets (HFD) because of their influence on health [3]. While studying the dissimilar effect of fats with different degrees of saturation, we have previously reported several studies comparing the influence of virgin olive oil (EVOO) and butter (BT) on the intestinal microbiota of mice, both using genotyping methods [4] and massive sequencing [5]. In these works, we presented evidence supporting a link between specific diets, physiological parameters and some bacterial taxa. Moreover, by comparing EVOO and refined olive oil (ROO) diets, the possible effect of virgin olive oil polyphenols was uncovered [6]. These new culture-independent technologies can, however, address only global taxa, giving no discriminative data on how the different strains of a certain bacterial group are evolving in response to the diet, even though this strain-level change is most probably the first one to occur in the bowels. Intestinal microorganisms will deal with the capacity of self-regulation of the system, the available food and the competition for resources, and will have to defend themselves against chemical products or aggressive proteins, which will lead to many of these bacterial groups to develop strategies and defence mechanisms. Among these mechanisms, we can find the expression of virulence factors or antibiotic resistance. These defensive weapons will inescapably affect the host, potentially causing serious clinical consequences.

One of the best-studied genera in this sense is the genus *Enterococcus*, in which several of these factors are known. Enterococci are intestinal bacteria widely known for their presence both in food and the oral-faecal route, and their utilization and safety remain a source of controversy [7]. As lactic acid bacteria, they have been important in food fermentations [8] and are also used as probiotics [7]. However, they are important nosocomial pathogens too, which prevents them from getting their GRAS (Generally Recognized as Safe) qualification [9]. This is worsened by their ability to survive adverse environmental conditions and heat treatments [10], which makes enterococci a widely distributed microbial group [11]. Enterococcal strains from food [12], environmental or clinical origins [13] are then very carefully studied to evaluate their safety.

*Enterococcus* is therefore a good model of how certain diets can protect the host, promoting, or not, the growth of strains with different levels of safety once they have reached the intestine. Considering the antimicrobial effect of olive oil on bacterial taxa in vitro [14] and, in a murine model [6], it is also interesting to evaluate its potential role, when included in the diet, in selecting the strains that are going to thrive in the intestines of the host. Consequently, our objective has been to carry out a study on the safety level of enterococcal strains, isolated from faeces of mice fed with standard chow or enriched with EVOO, ROO or BT, by evaluating their antibiotic resistance, virulence factors, and the production of biogenic amines, as well as to ultimately determine if there are statistically significant differences among these enterococci isolated from the four different diets.

## 2. Results

### 2.1. Isolation and Identification of Bacterial Strains

**Figure 1:**
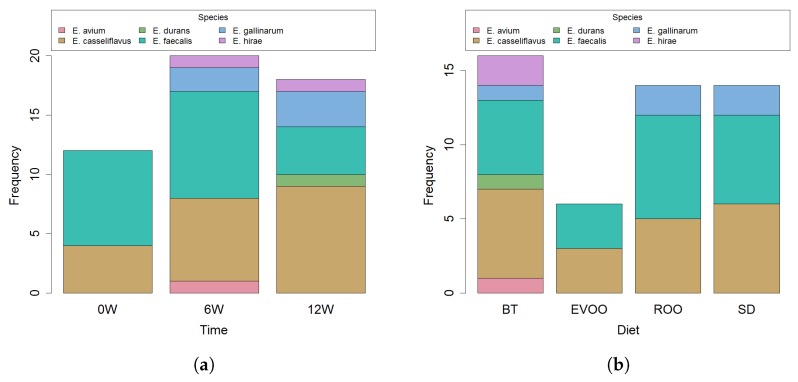
Bar plot of number of strains isolated within each species distributed according to time (**a**) and diet (**b**).

A previous collection of bacterial strains obtained from faeces of mice fed four different diets (SD, BT, EVOO and ROO) over a twelve-week period was screened for enterococcal phenotypic characteristics. All Gram positive cocci, facultative anaerobic, catalase negative, able to hydrolyse esculin in 40% bile salts and to grow from 10 to 45 ${}^{\circ }$C, in a media containing 6.5% NaCl or buffered at pH 9.6, were selected and their r16S gene was sequenced. In total, a collection of 50 enterococcal strains was obtained. As shown in Table 1, most of them belonged to the species *E. faecalis* [15] and *E. casseliflavus* [16], followed by *E. gallinarum* [5], *E. hirae* [2] and one strain of *E. avium* and *E. durans* each. Twelve strains were obtained at the beginning of the experiment, eighteen after six weeks and twenty strains after twelve weeks. Most strains were obtained in the BT group [17] and the lowest number of strains was found in the EVOO group [6], mostly because no strains were isolated from faeces on this diet at the end of the experiment (Figure 1a,b). To confirm this result statistically, a Poisson’s regression model was applied to the number of strains obtained based on two factors: diet and time (6 and 12 weeks). According to this model, the diet enriched with EVOO produced a significantly lower number of strains (23% lower; p=0.0221). However, in spite of these dissimilarities, no significant difference was found in the species distribution of the strains with respect to diet or time when applied a Fisher’s exact test for count data.

### 2.2. RAPD Classification

**Figure 2:**
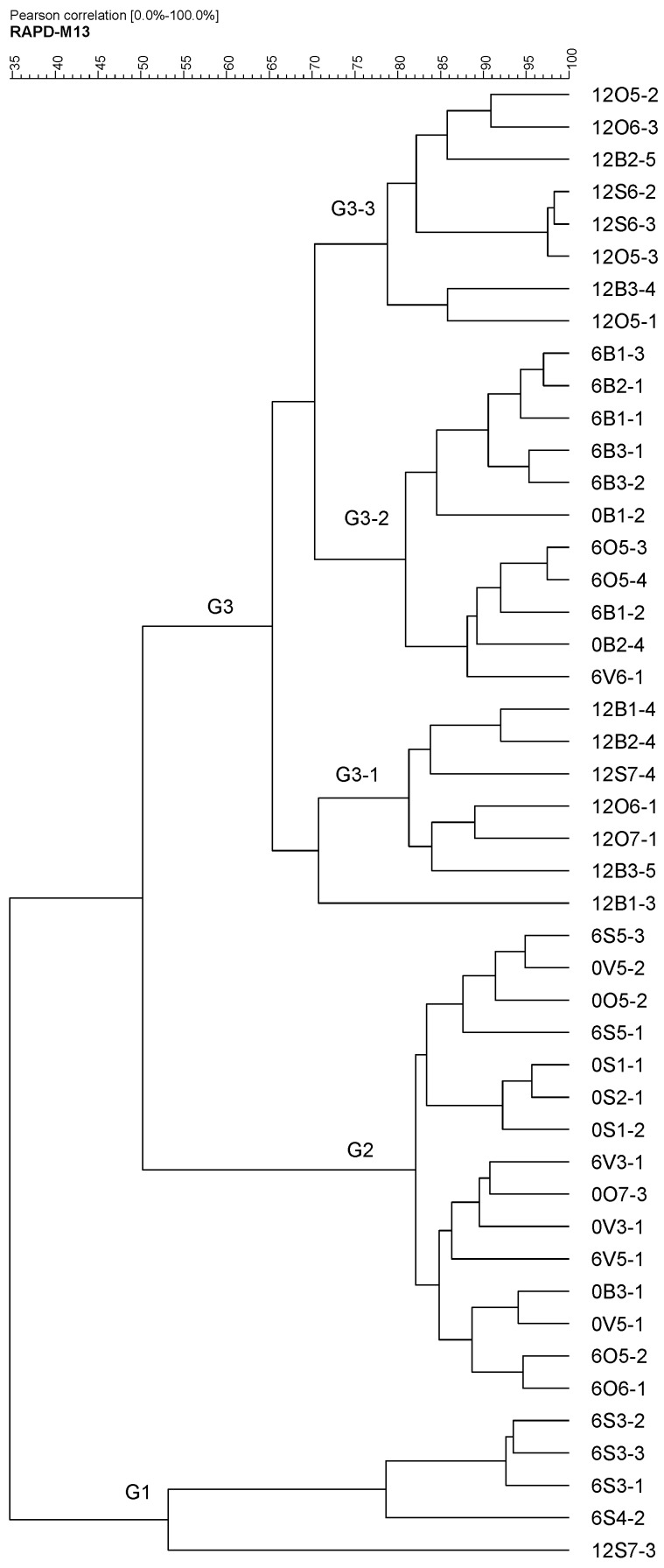
Pearson coefficient-based analysis of the RAPD profiles of the strains isolated. The initial number indicates the week of isolation.

When subjected to RAPD-PCR genotyping, the fifty strains clustered in three different groups (Figure 2). Group 1 was deeply branched and included only five strains, all of which were isolated from standard diet fed mice. Group 2 contained 40% of the isolates, all of them from the SD group or from the other groups at t=0. Finally, the third big cluster grouped the rest of the strains, 52%, most of them (21 out of 26) isolated from mice fed one of the three high fat diets (BT, EVOO or ROO).

### 2.3. PCR Amplification of Virulence Factors

Specific PCR reactions were performed to detect the presence of genes related to virulence factors (Table 1). Thirty-six isolates presented at least one virulence-related gene, out of which, six strains showed positive for two virulence factors and only one isolate presented three. Five strains showed positive for the aggregation substance gene (*agg*). Gelatinase gene (*gelE*) was amplified in only one isolate. Sex pheromone genes (*cpd* and *ccf*) were present in one and four strains, respectively, while *cob* was not detected. Of the cytolysin genes, only *cylB* was present, in only one strain, and genes related to cell wall adhesions (*efaAfs* and *efaAfm*) were detected in two strains each. In contrast, the gene coding for the enterococcal surface protein (*esp*) was present in most of the strains (62%).

**Figure 3:**
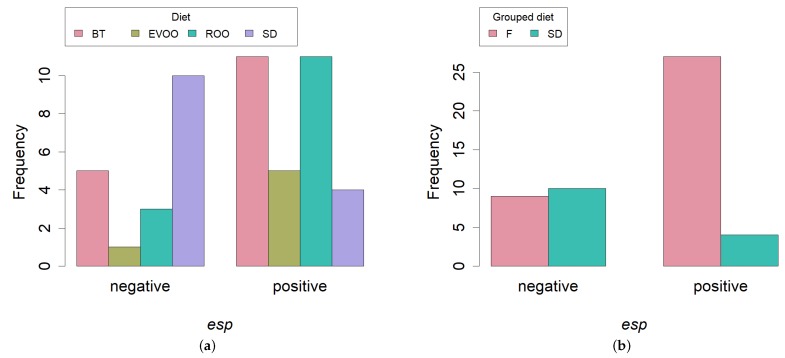
Bar plot of number of strains with presence (positives) or absence (negatives) of the gene *esp*, distributed among the four diets; (**a**) Fisher’s exact test p=0.0234) or between SD and high fat diets; (**b**) Fisher’s exact test p=0.0037). SD, standard diet; BT, butter enriched diet; EVOO, extra virgin olive oil enriched diet; ROO, refined olive oil enriched diet; F, all high fat diets.

Since the rest of virulence factors did not reach the number of positives needed for any statistical study, we centered our attention in the *esp* gene distribution. Contingent tables followed by Fisher’s exact test were performed to compare the percentage of presence of the gene in the strains isolated at the three different time-points and between each two different time-points, but no signification was detected. On the contrary, we found signification when comparing in the same way the percentage of presence of *esp* in the strains among all diets (p=0.0234; Figure 3a), with the lowest values obtained in the SD group. This difference turned out to be more significant when comparing the SD diet versus the three high fat diets (p=0.0037; Figure 3b).

### 2.4. Biogenic Amine Production

**Figure 4:**
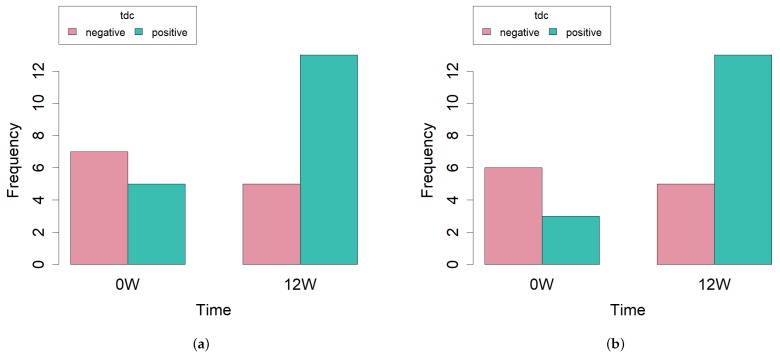
Bar plot of the number of strains with presence (positives) or absence (negatives) of the gene *tdc*, at the beginning or the end of the experiment considering strains from all diets; (**a**) Fisher’s exact test p=0.1362) or from all but an extra virgin olive oil enriched diet; (**b**) Fisher’s exact test p=0.0968).

Possible production of biogenic amines was evaluated through PCR amplification of the genes coding for the corresponding carboxylases. No amplification products of the genes *hdc1*, *hdc2* or *odc* were obtained. However, 29 strains were positive in the case of the gene coding for tyrosine decarboxylase (*tyr*) (Table 1). Again, contingent tables followed by Fisher’s exact test were performed in order to uncover differences among diets and also among all time-points. No significant differences were found in any case. When studying diets, a *p*-value of 0.4192 was obtained. When comparing data distribution between initial and final time points, the *p*-value, though still not significant, was lower (p=0.1362) with five and thirteen positive strains, respectively (Figure 4a). Since there were no strains isolated in the EVOO group at 12 weeks, we lacked one time-point in the contingency table with respect to his diet. In fact, we have shown above that, on this diet, the number of strains is significantly smaller. Given that this could introduce an additional factor not relevant for the question asked and that this produces a sparse contingency table, we decided to repeat the statistical analysis considering only those diets that present data along the three time points (SD, BT and ROO). In this case, the *p*-value (p=0.0968) is significant at 90% (Figure 4b).

### 2.5. Antibiotic Resistance

**Figure 5:**
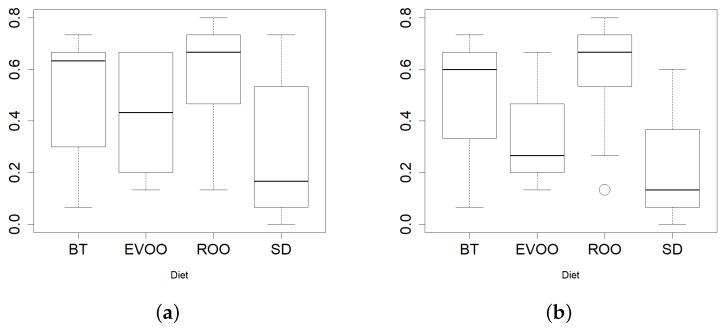
Boxplots of percentage of resistances in each strain grouped by diet considering all time-points; (**a**) p SD vs. ROO = 0.0225) or without initial time data; (**b**) p SD vs. ROO = 0.0039).

Resistance level was tested for fifteen different antibiotics and results were marked as R (resistance), I (intermediate) or S (susceptible) (Table 2). No antibiotic presented the same effect on all the strains; on the contrary, there was a high variability except in the case of kanamycin, norfloxacyn and levofloxacin, where most strains were sensitive, with only five resistant strains against the first antibiotic and six strains against each of the other two. Thirty-one strains were resistant to five or more antibiotics, and three isolates had no resistance at all. Then, the percentage of antibiotics to which each strain was resistant was calculated and these data were grouped by diet and by time-point and were compared using a Kruskal–Wallis test. No significant differences were found among the three time-points, but there was significance when the diets were compared (p=0.0225; Figure 5a). A pair comparison uncovered the fact that strains isolated from mice under SD diet presented less resistances than any of the three high fat diets, this difference being statistically significant when compared with the ROO diet (p=0.0140). If the initial time point was not considered, the significance of the difference with the ROO diet improved (p=0.0039), even reaching 90% significance also in the comparison between SD and BT (p=0.0804; Figure 5b).

Results on the genetic determinants of resistance for the isolates are shown in Table 3. All strains but one tested positive to at least one genetic determinant studied. Among the β-lactamase tested, *bla*${}_{PSE}$ and *bla*${}_{TEM}$ were the most frequent with presence in 23 and 24 strains, respectively, while *tetE* was the most prominent tetracycline resistance gene with 26 positives. Additionally, 34 strains presented genes for efflux pumps.

### 2.6. Growth with Virgin and Refined Olive Oil

Refined or virgin olive oil spotted directly on the lawn prevented any bacterial growth. Therefore, in order to see differences in the antimicrobial effect between both oils, we tried to emulate physiological conditions and made emulsions of the oils with cholic acid, lecithin or a mixture of both. Under these conditions, there was no total inhibition in any case and bacterial growth or partial inhibition alternated in all diets and with both types of oil (Table 4). When contingency tables were performed with these two options (growth/partial inhibition) and the four diets, no significant differences were detected among them.

**Figure 6:**
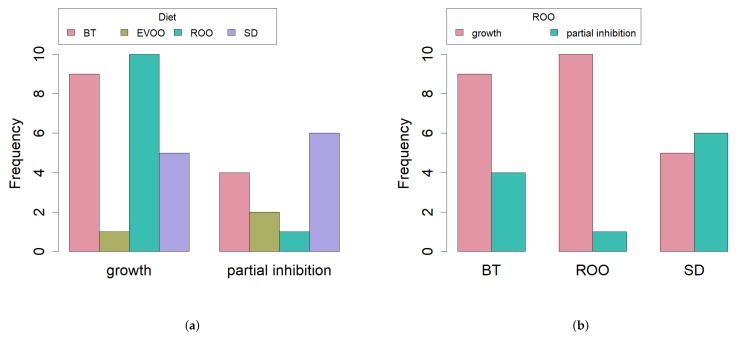
Bar plots of the number of strains with full growth or partial inhibition under refined olive oil; (**a**) according to diet (Fisher’s exact test p=0.0692); (**b**) paired data analysis of strains isolated from BT (McNemar’s test p=0.0040), ROO (McNemar’s test p=0.0019) and SD (McNemar’s test p=0.062). SD, standard diet; BT, butter enriched diet; EVOO, extra virgin olive oil enriched diet; ROO, refined olive oil enriched diet.

When the antimicrobial effect of EVOO and ROO was checked with the filter paper technique, we found partial inhibition in all strains when confronted with virgin olive oil but growth/partial inhibition alternated again with refined olive oil (Table 4). The highest percentage of unaffected strains/the lowest percentage of partial inhibition was found in the group of isolates from the ROO diet (Figure 6a,b). Again, a contingency table was performed to check whether distribution of data among the diets was or not homogeneous, and these time differences were found at 90% of signification (p=0.0692), confirming a strong tendency (Figure 6a).

Finally, each diet data were studied independently by means of a paired data analysis, with the McNemar’s test and exact *p*-values, in order to check signification of the differences in growth/partial inhibition when facing refined or virgin olive oil with the different techniques. This analysis could not be performed in the strains isolated from the EVOO diet because of its low number. With respect to the other three diets, no significant differences were found in the case of lecithin, cholic acid or the mixture of both. However, when studying data using filter paper (Figure 6b), significant differences were found for the ROO diet isolates (p=0.0019) and for the BT diet isolates (p=0.004), indicating a significantly lower number of partially inhibited strains when growing with refined olive oil with respect to growing with virgin olive oil in both cases. However, no significant differences at 95% were found for the SD diet isolates (p=0.062), indicating a higher number of partially inhibited strains.

## 3. Discussion

Since the development of culture-independent methods and massive sequencing technologies, the number of publications about gut microbiota and the effect of diets on the intestinal bacterial taxa has been ever increasing. This phenomenon has definitely been fuelled by the straightforwardness and high coverage of next generation sequencing, but this technique also has the drawback of only being reliable in reaching taxon levels of genera and above. However, many of the traits important in food safety and human health are species or even strain specific. Therefore, it is important to turn back to culture-dependent techniques and strain characterization in order to address how diet and other possible factors affect strain selection and population dynamics in an intestinal environment.

One of the best bacterial groups to study these influences in is the genus *Enterococcus*. Enterococci are intestine dwellers [10], able to survive in varied environmental conditions and widely present in food and food fermentations [11]. They have coevolved with humans and are symbionts with us but can also bear important virulence factors and become dangerous nosocomial germs. It is therefore an interesting model to study how the organism copes to coexist with such a variety of beneficial and detrimental strains of the same species, presumably managing to select those that are more advantageous.

In this research work, a collection of enterococci isolated from mice fed standard chow or three fat-enriched diets were studied and evaluated for safety. The most evident result was the inability to isolate any strain from the EVOO fed mice after twelve weeks of diet, which gave place to a statistically significant lower total number of strains isolated from this group. This decrement did not correspond to a statistically different distribution of the species in any diet or time-point; we did not find differences in the species distribution probably due to the fact that all but two of the species found in our study were represented by a low number of isolates.

When studying the genetic diversity of the strains on the basis of a RAPD-PCR genotyping, three main groups of strains were found. Interestingly, all the strains isolated from HFD fed mice clustered together in G3. This group divided into three subgroups. Groups G3-1 and G3-3 included only strains isolated after twelve weeks of experiments, while G3-2 only had strains isolated after six weeks. Groups G1 and G2, on the contrary, comprised strains isolated from the SD fed group or from the other three groups but at the beginning of the experiment, before HFD were applied. This clustering indicates already a clear genotypic separation between SD and HFD and, additionally within the latest, between six and twelve weeks of HFD application.

Most of the strains (31) were positive for the gene encoding the enterococcal surface protein (*esp*), which is not surprising in strains from a faecal origin, since this protein is known to promote the primary attachment to biotic surfaces and to be involved in immune system evasion [18]. However, unexpectedly, the presence of the gene was significantly different in the various diets, this difference being greatest when comparing a standard diet against HFD fed mice. This could uncover the existence of a selecting force in those intestines with a high fat input in such a way that only the enterocci with an increased efficiency in their attachment to the host mucosa can thrive. HFD would therefore prove to have promoted the presence of one virulence factor and, in any case, it is further evidence of how diet can select the types of strains that live within the host.

The presence of decarboxylases is another undesired trait in enterococci in foods since they produce biogenic amines through amino acid decarboxylation [19] and ingestion of high amounts of these compounds can cause varied toxicological hazards [20]. In our strains, the only decarboxylase gene found was *tyr*, coding for tyrosine decarboxylase, which is very commonly found in food commodities [21,22]. No differences were found in the presence of *tyr* in the diverse diets, but there was a strong tendency to increase with respect to time, with signification at 90%. This tendency is worth considering further since it could be a trait related to the age of the host, with implications for the host’s health since tyramine has been linked to hypertension and to an increment in glucose in serum [23].

When we set up the collection of strains to grow under a filter paper soaked in virgin olive oil, all of them were partially inhibited. However, when refined olive oil was used instead, a variable number of strains grew at a normal pace, showing no inhibition. This allowed us to make comparisons among the four groups of isolates from the different diets, uncovering a slight difference in the growth/partial inhibition distribution between diets, the strains isolated from ROO-fed mice being the least inhibited.

However, the most interesting result was obtained after searching for statistical signification in the growth behaviour of the strains within each diet. This would allow us to significantly state if the group of strains isolated from a certain diet was partially inhibited or not by refined olive oil, always in comparison to the partial inhibition exerted by virgin olive oil in all strains. A paired data analysis clearly stated that strains isolated from ROO and BT fed mice grew better in the presence of refined olive oil than in the presence of virgin olive oil, while strains isolated from standard fed animals were equally inhibited by both oils. This result is noteworthy since it could indicate an adaptation of the first two groups of strains to a fat-enriched environment, an adaptation that is, however, less useful when faced with virgin olive oil, where other antimicrobial factors are present in its unsaponifiable fraction, i.e., polyphenols [6,14,24].

To summarise, in this specific study, we have shown the influence of the type of diet on the selection of the virulence traits of the strains that thrive within the host. In the case we present, high fat diets seem to enhance the presence of a virulence trait (*esp*), increase the number of antibiotic resistances and improve the strain’s adaptation to grow in a fatty environment. However, virgin olive oil appears once more to behave differently probably due to the additional antimicrobial activity conferred by its minority components, which eventually might have an effect on the intestinal enterococci. Although framed in a very specific experimental model, these are interesting preliminary data that open new research lines. More studies using wider cohorts and categories will reinforce and delimitate these results.

## 4. Materials and Methods

### 4.1. Isolation and Identification of Bacterial Strains

The bacterial strains used in this study belong to a collection obtained in a previous work [4]. Briefly, as described previously [4], twelve male Swiss Webster ICR (CD-1) mice (Harlan Laboratories) were divided into four groups and fed for three months with a standard chow (SD, standard laboratory mice diet A04, 3% fat, Panlab, Barcelona, Spain) or one of three high fat diets (standard chow supplemented with 20% of extra-organic virgin olive oil—EVOO, refined olive oil—ROO, or butter—BT). All experimental procedures were performed in accordance with the European Communities Council Directive 86/609/EEC and reviewed and approved by the Bioethics Committee of the University of Jaén. In the first, sixth and twelfth week of the experimental period, faecal samples were obtained and serial dilutions of faeces were used for plating on Tryptic Soy Agar (Scharlab, Barcelona, Spain) plus 100 mg/L polymyxin B, and on Bile Esculin Agar (Scharlab), in order to select for enterococci colonies.

Putative enterococci were selected according to observation of colony characteristics and cell morphology, Gram staining, catalase and oxidase production, growth at 10 ${}^{\circ }$C and 45 ${}^{\circ }$C, growth in the presence of 6.5% NaCl, at pH 9.6 and growth and esculin hydrolysis on bile-esculin agar (Scharlab). Genetic identification at species level was done by species-specific PCR and 16S rDNA sequencing as described elsewhere [25].

### 4.2. RAPD-PCR Amplification

Once the strain DNA was extracted, genotyping using randomly amplified PCR was carried out using the primers M13 and AP4 as described elsewhere [8]. The RAPD patterns obtained with all strains and each one of the primers were analyzed in a single dendrogram using BioNumerics software version 2.5 (Applied Maths, Kortrijk, Belgium) as also indicated in the above reference.

### 4.3. PCR Amplification of Virulence Factors

Specific PCR reactions were performed in duplicate to detect the presence of genes involved in the expression of cytolysin (*cylA*, *cylB* and *cylM*), the aggregation substance (*agg*), gelatinase (*gelE*), enterococcal surface protein (*esp*), cell wall adhesions (*efaAfs* and *efaAfm*), and sex pheromones (*cpd*, *cob* and *ccf*), as established by Eaton and Gasson [17]. The positive control used was the DNA of *E. faecalis* P9091, since it contains in its genome all the virulence factors. In order to prove primer specificity, possible amplicons were checked by PCR simulators using *Enterococcus* and Bacteria genomic databases as templates (insilico.ehu.es and Primer-BLAST NCBI). This is also applicable to Section 4.4 and Section 4.5.

### 4.4. Biogenic Amine Production

The potential for biogenic amine generation was detected by PCR amplification in duplicate of the amino-acid decarboxylase genes *hdc* (histidine decarboxylase), *odc* (ornithine decarboxylase) and *tdc* (tyrosine decarboxylase) as described before [26].

### 4.5. Antibiotic Resistance

The antibiotic susceptibility of isolates was determined using ATB ENTEROC 5 strips (BioMérieux, Marcy-l’Etoile, France), following the manufacturer’s instructions. The 50 isolates were also investigated by PCR for the presence of genetic determinants of resistance as well as presence of efflux pumps. Extended-spectrum β-lactamase: *bla*${}_{CTX-M}$ and *bla*${}_{CTX-M2}$ [27], *bla*${}_{PSE}$ [28] and *bla*${}_{TEM}$ [16], groups I and II tetracycline resistance genes [15], and efflux pumps *acrB*, *mdfA* [29] and *aadA1* [30] were tested.

### 4.6. Growth with Virgin and Refined Olive Oil

The antimicrobial effect of virgin and refined olive oil on the strains was measured by two methods. In the first one, the spot-on-a-lawn method, 150 μL of overnight culture was plated per triplicate on Brain Heart Infusion agar (Scharlab) and 5 μL axenic spots were added each containing one of the following: virgin olive oil; refined olive oil; a 50:50 mixture of 1% cholic acid and virgin or refined olive oil; a 50:50 mixture of 3% lecithin and virgin or refined olive oil; a 50:50 mixture of 1% cholic acid-3% lecithin and virgin or refined olive oil. Plates were incubated for 24 h at 37 ${}^{\circ }$C and microbial growth was evaluated. For the second method, filter fragments were used. Again, 150 μL of overnight culture was plated per triplicate on Brain Heart Infusion agar (Scharlab) and sterile 2 cm${}^{2}$ Whatman filter fragments soaked in saline solution, virgin olive oil or refined olive oil were laid on top. Plates were incubated for 24 h at 37 ${}^{\circ }$C and microbial growth was evaluated underneath. Results were defined as “growth”, “partial inhibition” or “total inhibition”. Partial inhibition was defined as that situation in which the surface covered by the drop or the filter does not present a continuous lawn of bacterial growth.

### 4.7. Statistical Studies

The number of animals in this study (three per diet) was determined in a previous work [4] where all animals on the same diet had been stabled in the same cage until the moment faeces were collected. In order to overcome the possibly low number of subjects, in this study, strains were taken as sampling units and contingency tables were designed where each cage at a certain time-point was the basis of a different category of the variable “diet”.

To test whether or not the variable “species” has the same distribution in the different categories of the variable “diet”, and in the different categories of the variable “time”, the exact Fisher’s test for contingency tables was performed as some of the expected counts were below 5 and therefore the $\chi^{2}$ test of homogeneity is not suitable. Similar analyses were carried out to identify whether there was an influence of “diet” or “time” in the variables *tdc* and *esp*, as well as to study whether “diet” had an influence on “growth/partial inhibition” on the different oils and procedures.

To investigate the effect of the variables “diet” and “time” on the number of enterococci strains, a Poisson regression model was fitted. As “diet” and “time” are categorical variables, dummy variables were included in the model in order to evaluate the effect that the presence or absence of the different levels of the categorical variables may have on the number of strains.

To test equality of the distribution of the variable “% of antibiotic resistances” according the type of diet we used the Kruskal–Wallis test as the Shapiro–Wilk test of normality rejected the normality hypothesis. The same procedure was performed to study the equality of the distribution of the variable “% of antibiotic resistances” according to the variable “time”. Provided that significant differences were detected, we applied post hoc Duncan tests for pairwise multiple comparisons with Bonferroni’s adjustment in the *p*-values.

To study whether or not there are differences in the antimicrobial effect of ROO and EVOO within each specific diet, only the growth/partial inhibition of the strains isolated from that group of mice was observed. As the results are paired nominal data, the McNemar test was used to check the statistical significance.

## Figures and Tables

**Table 1:** Detection of virulence factors and biogenic amines gene products.

		Virulence Factors											Biogenic Amines
	**Species**	***agg***	***gelE***	***cylA***	***cylM***	***cylB***	***esp***	***efaAfs***	***efaAfm***	***cob***	***cpd***	***ccf***	***tdc***
0S1-1	*E. faecalis*	+											
0S1-2	*E. casseliflavus*	+											
0S2-1	*E. faecalis*	+											+
0B1-2	*E. casseliflavus*						+						
0B2-4	*E. faecalis*						+						
0B3-1	*E. casseliflavus*						+						
0O5-2	*E. faecalis*						+					+	+
0O5-4	*E. faecalis*						+						+
0O7-3	*E. faecalis*						+						
0V3-1	*E. casseliflavus*						+						
0V5-1	*E. faecalis*						+						+
0V5-2	*E. faecalis*						+					+	+
6S3-3	*E. casseliflavus*					+		+	+				
6S3-4	*E. gallinarum*												+
6S4-1	*E. casseliflavus*												
6S4-2	*E. faecalis*						+					+	
6S5-1	*E. faecalis*												+
6S5-3	*E. faecalis*		+				+						+
6B1-1	*E. casseliflavus*						+						
6B1-2	*E. gallinarum*											+	
6B1-3	*E. hirae*						+						+
6B2-1	*E. faecalis*						+						+
6B3-1	*E. avium*						+						+
6B3-2	*E. casseliflavus*						+						
6O5-2	*E. faecalis*						+						+
6O5-3	*E. faecalis*												+
6O5-4	*E. faecalis*						+						+
6O6-1	*E. faecalis*						+						+
6O7-1	*E. casseliflavus*						+						
6V3-1	*E. casseliflavus*						+						
6V5-1	*E. faecalis*								+				+
6V6-1	*E. casseliflavus*	+					+						
12S6-2	*E. faecalis*												+
12S6-3	*E. casseliflavus*										+		+
12S7-2	*E. casseliflavus*												+
12S7-3	*E. gallinarum*						+						+
12S7-4	*E. casseliflavus*						+						+
12B1-3	*E. hirae*												+
12B1-4	*E. faecalis*						+	+					+
12B2-4	*E. casseliflavus*						+						+
12B2-5	*E. durans*												
12B3-2	*E. casseliflavus*						+						
12B3-4	*E. faecalis*												+
12B3-5	*E. faecalis*												+
12O5-1	*E. gallinarum*	+					+						
12O5-2	*E. casseliflavus*						+						
12O5-3	*E. gallinarum*												+
12O6-1	*E. casseliflavus*						+						+
12O6-3	*E. casseliflavus*												+
12O7-1	*E. casseliflavus*						+						

**Table 2:** Antibiotic resistance. R: Resistant I: Intermediate S: Susceptible. Antibiotics. PENP: penicillin pneumo; PENS: penicillin strepto; AMPE: ampicillin; CTXP: cetofaxim pneumo; CTXS: cetoaxim strepto; IMIE: imipenem; KAHES: kanamycin; GEHES: gentamycin; FQPR: norfloxacin; MXFPS: moxifloxacin; LVXS: levofloxacin strepto; LVXP: levofloxacin pneumo; ERYPS: erythromycin; TELPS: telithromycin; QDAE: quinupristin-dalfopristin; TEPS: tetracyclin; RFAPS: rifampicin; TSU: cotrimoxazol; LNZEP: linezolid pneumo; LNZS: linezolid strepto; FOSP: fosfomycin; FURES: nitrofurantoin; VAN: vancomycin; TEC: teicoplanin.

	Antibiotic Resistance														
	**PEN**	**PENS**	**AMPE**	**CTXP**	**CTXS/IMIE**	**KAHES/GEHES**	**FQPR/MXFPS**	**LVXS/LVXP**	**ERYPS/TELPS**	**QDAE**	**TETPS/RFAPS**	**TSU/LNZEP**	**LNZS**	**FOSP7FURES**	**VAN/TEC**
0S1-1	R	R	I	R	I	S	S	S	R	R	R	R	R	I	R
0S1-2	R	R	I	R	I	S	S	S	R	R	I	I	R	S	R
0S2-1	R	R	R	R	I	S	S	S	R	R	R	R	R	R	R
0B1-2	I	S	S	R	I	S	S	S	S	S	S	S	S	S	S
0B2-4	R	R	R	R	I	S	S	S	R	R	R	R	R	R	R
0B3-1	R	R	R	I	I	S	I	S	R	R	R	R	R	R	R
0O5-2	R	R	R	R	R	S	S	S	R	R	R	I	R	R	R
0O5-4	R	I	S	R	I	S	S	I	S	I	R	S	I	I	S
0O7-3	R	R	I	R	I	S	S	S	R	R	R	I	R	I	R
0V3-1	R	R	R	I	I	S	S	S	R	R	R	I	R	R	R
0V5-1	R	R	R	R	I	S	S	S	R	R	R	I	R	R	R
0V5-2	I	I	S	R	I	S	S	S	I	R	R	S	S	S	S
6S3-3	R	R	I	I	I	S	S	S	I	S	S	S	S	S	S
6S3-4	I	S	S	I	I	S	S	S	S	S	S	S	S	S	S
6S4-1	R	R	I	R	I	S	S	S	R	R	R	I	R	S	R
6S4-2	R	R	S	R	I	S	S	S	I	S	S	S	S	S	S
6S5-1	I	I	S	I	I	S	S	S	I	I	I	S	S	I	S
6S5-3	I	I	S	S	S	S	S	S	I	I	R	S	S	I	S
6B1-1	R	I	S	R	I	I	S	S	I	R	R	S	S	I	S
6B1-2	R	R	R	R	I	S	I	S	R	R	R	I	R	R	R
6B1-3	I	S	S	R	I	S	S	S	I	I	I	S	I	S	S
6B2-1	R	R	R	R	R	R	R	R	R	S	I	I	S	S	S
6B3-1	R	R	R	R	I	S	R	S	R	R	R	I	R	R	R
6B3-2	R	I	S	R	I	S	S	S	R	R	R	S	S	I	S
6O5-2	R	R	R	I	S	S	S	R	R	R	R	I	R	R	R
6O5-3	R	R	R	R	I	S	S	S	R	R	R	I	R	R	R
6O5-4	R	R	R	R	R	S	S	S	R	R	R	I	R	R	R
6O6-1	R	R	R	R	I	S	S	S	R	R	R	I	R	R	R
6O7-1	R	I	S	R	I	S	S	S	I	R	R	S	S	S	S
6V3-1	R	R	R	R	I	S	I	S	R	R	R	I	R	R	R
6V5-1	I	I	S	R	I	S	S	S	R	R	R	S	S	I	S
6V6-1	I	I	S	I	I	S	S	S	I	R	R	S	S	S	S
12S6-2	R	R	I	S	S	S	S	S	R	R	R	R	R	I	R
12S6-3	I	I	S	R	I	S	S	S	I	I	I	S	S	I	S
12S7-2	I	I	S	R	I	S	S	S	I	I	I	S	S	I	S
12S7-3	R	R	S	I	S	S	S	S	I	S	S	I	S	S	S
12S7-4	R	R	I	R	I	S	S	S	R	R	R	R	R	S	R
12B1-3	R	R	R	R	R	R	R	R	R	S	R	I	S	S	S
12B1-4	R	R	R	R	R	R	R	R	R	S	R	I	S	S	S
12B2-4	R	I	S	R	I	S	S	S	I	R	R	S	S	R	S
12B2-5	R	I	S	R	I	S	S	S	I	R	R	S	S	R	S
12B3-2	R	R	R	R	R	R	R	R	R	S	R	I	S	S	S
12B3-4	R	R	R	R	R	R	R	R	R	I	R	S	S	I	I
12B3-5	I	I	S	I	I	S	S	S	I	S	R	S	S	I	S
12O5-1	R	R	S	R	I	S	S	S	R	R	R	I	R	I	S
12O5-2	R	R	R	R	R	S	R	I	R	R	R	I	R	R	R
12O5-3	I	S	S	R	I	S	S	S	S	S	R	S	S	I	S
12O6-1	R	R	R	R	R	S	S	S	R	R	R	I	R	R	R
12O6-3	R	R	R	R	I	S	S	S	R	R	R	I	R	S	R
12O7-1	R	R	R	R	R	S	S	S	R	R	R	I	R	R	R

**Table 3:** Genetic determinants of resistance.

	β-Lactamases				Tetracyclines						Efflux Pumps		
					**Group I**			**Group II**					
	***bla*** ${}_{\mathrm{CTX}-M}$	***bla*** **${}_{\mathrm{CTX}-M2}$**	***bla*** ${}_{\mathrm{PSE}}$	***bla*** ${}_{\mathrm{TEM}}$	***tetB***	***tetC***	***tetD***	***tetA***	***tetE***	***tetG***	***acrB***	***aadA1***	***mdfA***
0S1-1	+	+		+							+		+
0S1-2	+	+		+						+	+	+	+
0S2-1	+			+		+					+		+
0B1-2				+									
0B2-4			+						+				
0B3-1				+						+			
0O5-2			+					+	+				
0O5-4									+				
0O7-3	+			+						+	+	+	
0V3-1	+		+	+						+	+	+	
0V5-1									+		+	+	
0V5-2			+						+				
6S3-3		+	+					+	+		+		
6S3-4	+		+		+	+					+	+	+
6S4-1			+						+		+		
6S4-2		+	+					+			+		
6S5-1	+		+						+			+	
6S5-3	+	+						+	+		+		
6B1-1				+						+		+	
6B1-2			+						+	+			
6B1-3		+	+						+				
6B2-1	+		+	+					+				
6B3-1			+	+					+			+	
6B3-2		+		+							+	+	
6O5-2		+	+						+				
6O5-3	+	+	+						+				
6O5-4		+	+					+	+				
6O6-1			+						+		+	+	
6O7-1				+						+	+	+	
6V3-1				+								+	
6V5-1				+					+	+	+	+	+
6V6-1				+						+			
12S6-2											+		+
12S6-3	+			+		+		+			+	+	+
12S7-2											+		+
12S7-3		+	+					+			+		
12S7-4		+									+		+
12B1-3	+		+						+	+			+
12B1-4									+			+	
12B2-4	+		+						+				
12B2-5			+						+		+		
12B3-2	+			+					+	+	+	+	
12B3-4				+					+	+			
12B3-5													
12O5-1				+						+		+	
12O5-2				+						+		+	
12O5-3				+								+	
12O6-1	+			+					+	+	+	+	
12O6-3			+	+						+		+	
12O7-1			+	+					+	+	+	+	

**Table 4:** Refined (ROO) and virgin olive oil (EVOO) antimicrobial activity. PI: Partial inhibition.

	Cholic Acid 1%		Lecitine 3%		Lecitine 3% + Cholic Acid 1%		Filter Paper	
	**ROO**	**EVOO**	**ROO**	**EVOO**	**ROO**	**EVOO**	**ROO**	**EVOO**
0S1-1	PI		PI	PI				PI
0S1-2				PI				PI
0S2-1								PI
0B1-2								PI
0B2-4	PI	PI	PI	PI				PI
0B3-1			PI					PI
	**ROO**	**EVOO**	**ROO**	**EVOO**	**ROO**	**EVOO**	**ROO**	**EVOO**
0O5-2								PI
0O5-4			PI			PI		PI
0O7-3						PI		PI
0V3-1		PI						PI
0V5-1								PI
0V5-2	PI	PI		PI				PI
6S3-3				PI				PI
6S3-4								PI
6S4-1		PI		PI			PI	PI
6S4-2							PI	PI
6S5-1				PI				PI
6S5-3	PI			PI		PI	PI	PI
6B1-1								PI
6B1-2		PI						PI
6B1-3								PI
6B2-1	PI						PI	PI
6B3-1							PI	PI
6B3-2	PI	PI		PI			PI	PI
6O5-2	PI	PI	PI	PI		PI		PI
6O5-3	PI		PI					PI
6O5-4						PI		PI
6O6-1								PI
6O7-1								PI
6V3-1								PI
6V5-1						PI	PI	PI
6V6-1							PI	PI
12S6-2						PI	PI	PI
12S6-3							PI	PI
12S7-2								PI
12S7-3			PI				PI	PI
12S7-4								PI
12B1-3								PI
12B1-4								PI
12B2-4								PI
12B2-5								PI
12B3-2								PI
12B3-4						PI		PI
12B3-5							PI	PI
12O5-1		PI	PI	PI		PI		PI
12O5-2								PI
12O5-3						PI		PI
12O6-1						PI		PI
12O6-3								PI
12O7-1							PI	PI
